# Characterization and Encapsulation of Natural Antioxidants: Interaction, Protection, and Delivery

**DOI:** 10.3390/antiox11081434

**Published:** 2022-07-24

**Authors:** Hao Cheng, Li Liang

**Affiliations:** 1 State Key Laboratory of Food Science and Technology, Jiangnan University, Wuxi 214122, China; haocheng@jiangnan.edu.cn; 2 School of Food Science and Technology, Jiangnan University, Wuxi 214122, China

Natural antioxidants (e.g., polyphenols, carotenoids, vitamins, polyunsaturated fatty acids, peptides, and enzymes) are expected to inhibit the oxidative damage of proteins and lipids in food systems and reduce the risk of certain chronic diseases related to oxidative stress [1]. It is still a challenge to predict an antioxidant’s activity based on its structural characteristics, especially for peptides [2]. Furthermore, most of natural antioxidants have poor aqueous solubility, high sensitivity to processing and storage conditions, and low bioaccessibility and bioavailability, which restrict their direct incorporation into commercial products and their related health benefits [3].

According to a Market Research Report of Global Industry Analysis Inc., the global market for food antioxidants, which is estimated at USD 1.2 billion in 2020, is projected to grow at a CAGR of 3.5% and reach a revised size of USD 1.6 billion by 2027, while natural food antioxidants are projected to record 3.8% CAGR and reach USD 1.1 billion by 2027 [4]. It is well known that encapsulation technology plays an important role in protecting and stabilizing natural antioxidants [5]. Inorganic materials, proteins, polysaccharides and lipids are potential carrier materials for antioxidants. Many of their structural and physicochemical properties facilitate the design and fabrication of delivery systems in a wide range of platforms, which can overcome the limitations of natural antioxidants used in food and pharmaceutical industries [6,7,8].

This Special Issue contains 13 research articles covering the prediction of antioxidant peptides and the improvement of the aqueous solubility, stability, bioactivity and release properties through enzymatic glycosylation and encapsulation technology in addition to a review about ascorbic acid, which describes the bioactivity and degradation mechanism of ascorbic acid, the strategies for improving its chemical stability, and the application of ascorbic acid in commercial products [9].

The antioxidant activity of peptides derived from protein is usually attributed to their amino acid composition, active amino acid position, molecular mass, and spatial structure. How to directly screen antioxidant peptides according to their physicochemical properties is essential to the efficiently manufacturing of peptides with high antioxidant activity. Shi et al. confirmed that the route of a molecular weight of 200 to 800 Da containing Tyr, Met, and Trp residues at the C-terminus and a grand mean hydropathy value between −2 and 1 could be used to screen antioxidant peptides. They found that peptides released from hazelnut protein falling within the route could effectively prevent the oxidation of linoleic acid and hazelnut oil [10].

Mangiferin, a C-glucosidic xanthone from *Mangifera indica* (mango) plant, exhibits many health-promoting activities. However, the poor hydrosolubility of mangiferin limits its application in the food industry. In order to improve the aqueous solubility of mangiferin, the enzymatic glycosylation of mangiferin was applied to produce more soluble mangiferin glucosides. The recombinant maltogenic amylase was produced by cloning a thermophile *Parageobacillus galactosidasius* DSM 18751 T (PgMA) into *Escherichia coli* BL21 (DE3) via the expression plasmid pET-Duet-1. The obtained glucosyl-α-(1→6)-mangiferin and maltosyl-α-(1→6)-mangiferin from mangiferin by PgMA showed 5500-fold higher aqueous solubility than that of mangiferin. Both mangiferin glucosides showed similar DPPH free radical scavenging activities as mangiferin [11].

Inorganic nanoparticles, such as magnetic and selenium nanoparticles, can serve as antioxidants or delivery vehicles for bioactives. Mandić et al., synthesized mesoporous magnetite nanoparticles stabilized by PEG-4000 using a solvothermal method. The magnetite nanoparticles had a small size of around 10 nm and encapsulation efficiency of 20% for quercetin. The prolong quercetin release from PEG-coated magnetite nanoparticles could be realized by using combined stationary and alternating magnetic fields [12]. In another study, the *Polygonatum sibiricum* polysaccharide was used as a stabilizer of selenium nanoparticles in a simple redox system. The functionalization with the *Polygonatum sibiricum* polysaccharide effectively improved the free-radical-scavenging ability of selenium nanoparticles. The obtained polysaccharide-coated selenium nanoparticles showed a higher protective effect on PC-12 cells against H_2_O_2_-induced oxidative damage [13].

Protein particles with a hollow structure are preferred over solid ones due to their high loading capacity, sustained release, and low density. Khan et al. fabricated hollow zein particles through a sacrificial template method for the encapsulation of quercetin, obtaining a maximum encapsulation efficiency of 80% and loading capacity of 6.29%. The hollow zein particles were further coated with chitosan and pectin using a layer-by-layer technique, which improved the stability of hollow zein particles against heat treatment, pH variation, and salt. The obtained hollow zein composite particles could significantly improve the photostability and storage stability of quercetin [14]. Milea and coworkers prepared WPI-xylose Maillard-based conjugates for the encapsulation of flavonoids from yellow onion skins. The microcapsules formulated with WPI-xylose conjugates showed a high encapsulation efficiency of 90.53%. The incorporation of flavonoid-loaded microcapsules into nachos could improve the antioxidant activity of nachos [15]. Yin et al. investigated the interaction of whey protein isolate (WPI), sodium caseinate, and soy protein isolate with resveratrol, and they found that the protein species play an important role in loading and protecting antioxidants. WPI had the lowest loading capacity but showed the best protective effect for resveratrol against degradation during storage. The results highlight the importance of protein oxidability on stability [16].

Cyclodextrins with a hydrophobic interior and a hydrophilic outer surface can act as a carrier for phenolic compounds. Escobar-Avello et al. entrapped around 80% of the extracts from a grape cane in hydroxypropyl beta-cyclodextrin (HP-β-CD) through a spray-drying technique. The inclusion of grape cane extracts in HP-β-CD had no impact on their antioxidant activity [17]. Valentino and coworkers fabricated a thermo-responsive hydrogel with Pluronic F-127 and hyaluronic acid, which exhibited easy injectability and sol–gel transition as the temperature increased from room temperature to body temperature. The smart hydrogel was further used to encapsulate hydroxytyrosol-loaded chitosan nanoparticles for the treatment of osteoarthritis. The in vitro study demonstrated that the combination of hydrogels and antioxidant-loaded nanoparticles showed potential use in the treatment of the chronic inflammatory degenerative disease [18].

Lipid-based delivery systems, such as emulsions and liposomes, are effective carriers for enhancing the stability and bioavailability of bioactives [19]. Lemon essential oil with many biological activities is commonly used as a preservative or flavoring agent in the food industry. Liu and coworkers fabricated a stable nanoemulsion formulated with Tween-80 and Span-80 for the encapsulation of lemon essential oil. The optimal formulation was obtained by using a combination of single-factor experiments and response surface methodology. The lemon oil nanoemulsions exhibited improved antioxidant activity and stability during storage [20]. In another study, *Spirulina plantensis* protein hydrolysates were encapsulated into liposomes coated with chitosan. Around 90% of the initial antioxidant activity remained after storage at 4 °C for 30 days. The hydrolysate-loaded liposomes could be converted into powders by using a spray-dying technique, exhibiting all characteristics of peptide-loaded liquid formulations [21].

There are some examples of incorporating bioactive-loaded edible carriers into food products. Fish protein hydrolysates with high antioxidant and angiotensin-converting enzyme inhibitory capacity were microencapsulated into maltodextrin matrix using a spray-drying technique, which was further incorporated into yogurt products. Yogurts fortified with protein hydrolysates microcapsules have acceptable flavor and exhibited greater antioxidant and antihypertensive activities during a 7-day storage [22]. In another study, tamarillo polyphenols were successfully encapsulated in a cubosomal system. The addition of tamarillo polyphenol-loaded cubosome improved the physicochemical properties and nutritional values of yoghurts [23].

We would like to acknowledge all the authors who contributed to this Special Issue “Characterization and Encapsulation of Natural Antioxidants: Interaction, Protection, and Delivery”. This Special Issue provides new insights into the importance of delivery carriers in the stabilization and improved performance of natural antioxidants. Despite the relevance of the topics covered in the papers published in this Special Issue, many aspects remain relatively limited, including the mechanisms of antioxidant–material interplay, the compatibility of antioxidant-loaded carriers with commercial products, and the evaluation of bioactivity in vivo models.
